# Metagenomic comparison of the rectal microbiota between rhesus macaques (*Macaca mulatta*) and cynomolgus macaques (*Macaca fascicularis*)

**DOI:** 10.24272/j.issn.2095-8137.2018.061

**Published:** 2018-08-08

**Authors:** Yan-Fang Cui, Feng-Jie Wang, Lei Yu, Hua-Hu Ye, Gui-Bo Yang

**Affiliations:** 1 National Center for AIDS/STD Control and Prevention, China-CDC, Beijing 102206, China; 2 Laboratory Animal Center of the Academy of Military Medical Sciences, Beijing 100071, China

**Keywords:** Rhesus macaques, Cynomolgus macaques, Gut microbiota, Next generation sequencing

## Abstract

Rhesus macaques (*Macaca mulatta*) and cynomolgus macaques (*Macaca fascicularis*) are frequently used in establishing animal models for human diseases. To determine the differences in gut microbiota between these species, rectal swabs from 20 rhesus macaques and 21 cynomolgus macaques were collected, and the microbial composition was examined by deep sequencing of the 16S rRNA gene. We found that the rectal microbiota of cynomolgus macaques exhibited significantly higher alpha diversity than that of rhesus macaques, although the observed number of operational taxonomic units (OTUs) was almost the same. The dominant taxa at both the phylum and genus levels were similar between the two species, although the relative abundances of these dominant taxa were significantly different between them. Phylogenetic Investigation of Communities by Reconstruction of Unobserved States (PICRUSt) showed significant differences in the functional components between the microbiota of the two species, in particular the lipopolysaccharide (LPS) synthesis proteins. The above data indicated significant differences in microbial composition and function between these two closely related macaque species, which should be taken into consideration in the future selection of these animals for disease models.

## INTRODUCTION

Extensive studies have been conducted on the commensal bacteria in the gastrointestinal tract of mice and humans. The gut microbiota is well established as an integral part of the gut mucosal immune system (Backhed et al., 2005). Commensal bacteria not only affect the development and function of the gut mucosal immune system, but also that of the systemic immune system (Lathrop et al., 2011; Niess & Adler, 2010). Beyond the immune system, the physiologies of other functional systems, such as the nervous system, endocrine system and cardiovascular system, are also under the influence of the commensal microbiota (Rieder et al., 2017; Stilling et al., 2015). Many human diseases are associated with abnormality in gut microbiota or dysbiosis (Manichanh et al., 2006; Tilg & Moschen, 2014). Furthermore, infusion of microbiota from healthy donors has shown effects on gut diseases such as recurrent *Clostridium difficile* infection (van Nood et al., 2013). Therefore, studies on gut microbiota could shed light on our understanding of human health and disease.

Both rhesus macaques (*Macaca mulatta*) and cynomolgus macaques (*Macaca fascicularis*) have long been used in animal models of human diseases such as AIDS and diabetes (Bauer et al., 2011; Feichtinger et al., 1990; Letvin et al., 1985; Li et al., 2017). While the microbiotas of both rhesus and cynomolgus macaques have been reported previously, few comparative studies exist in the literature (Euler et al., 1990; Loftin et al., 1980). To characterize the gut mucosal immune system of macaques, we previously cloned the *MAdCAM-1*, *IL-22* and *DNGR-1* genes and examined their expression in the gut mucosa of rhesus macaques (Wang et al., 2014; Yao et al., 2017; Yu et al., 2018). In the current study, we examined the rectal microbiota of rhesus and cynomolgus macaques by deep sequencing of the 16S rRNA gene and subsequently analyzed their composition and predicted function.

## MATERIALS AND METHODS

### Sample collection

Rhesus macaques of Chinese origin and cynomolgus macaques of Indochinese origin (imported into China more than 20 years ago), 2–3 years old, were captive-bred and housed in the same experimental animal center in Beijing and fed the same diet (monkey chow plus seasonal fruit or vegetables). Stool samples were collected with cotton swabs from the rectum of 20 (10 males and 10 females) rhesus macaques and 21 (11 males and 10 females) cynomolgus macaques. All macaques were healthy and had no recent gastrointestinal abnormalities or abnormal weight changes at the time of sample collection. All samples were placed on ice immediately after collection and shipped to the laboratory and stored at −80 °C before use. All animals were treated humanely as approved by the Laboratory Animal Welfare & Ethics Committee of the China-CDC.

### Extraction of DNA

Total DNA was extracted from the stool samples using the QIAamp DNA Stool Mini Kit (Qiagen, Germany), following the manufacturer’s protocols. Briefly, 10–20 mg of each sample was lysed with buffer ASL, digested with protein kinase A, treated with buffer AL and loaded into a QIAamp spin column. After washing with AW1 and AW2, the bound DNA in the column was eluted into 50 μL of buffer AE. Aliquots of the extracted DNA samples were frozen at −80 °C before use.

### Polymerase chain reaction (PCR) and sequencing

For samples collected from each animal, the 16S rRNA gene was amplified from the extracted DNA using the primers R515F/806R. The PCR products were gel purified and the amplicons were quantified with Nanodrop 2000 (Thermo, USA). Equal amounts of DNA from each sample were mixed to construct the sequencing library and quantified by real-time PCR with the KAPA Library Quantification Kits (Illumina, USA). The DNA library of the 16S rRNA gene with 50% PhiX was sequenced on MiSeq with V3 sequencing reagent kits (Illumina, USA). FASTQ files were generated for each sample.

### Bioinformatic analysis

QIIME2 software package (version 2018.4) was used to clean the reads, calculate the alpha and beta diversities, and assign the taxonomy of each representative sequence according to the tutorial on the QIIME2 website (http://qiime2.org/). Greengenes (13_8) was used as a reference and 99% similarity was used for operational taxonomic unit (OTU) picking. The PICRUSt (Langille et al., 2013) and STAMP (Parks et al., 2014) software packages were used to analyze differences in microbial composition and function between the two species.

### Statistical analysis

For quantitative comparison of the diversities and compositions of the rectal microbiota between the two species, non-parametric statistical methods (Mann-Whitney *U* test or Wilcoxon sum rank test) were used. *P*-values less than 0.05 were considered statistically significant.

## RESULTS

Deep sequencing of the 16S rRNA gene in rectal stool samples from 20 rhesus and 21 cynomolgus macaques resulted in 668 486 clean reads for rhesus macaques (33 424 reads per sample), representing 957 OTUs, and 826 833 clean reads for cynomolgus macaques (39 373 reads per sample), representing 1 216 OTUs. In total 1 411 OTUs were identified in the 41 macaques.

### Alpha diversity of rectal microbiota in rhesus and cynomolgus macaques

To determine the microbial diversity differences in the microbiota between the two species, we examined the alpha diversity (level of diversity within individual samples) of the microbiota. As shown in Table 1, there were no differences in the observed OTUs and Chao 1 indexes between the microbiota of the two species. However, the Shannon, Simpson, Faith’s phylogenetic diversity (PD) and Good’s coverage indexes were significantly different between the two species.

**Table 1:** Alpha diversity of the rectal microbiota of rhesus and cynomolgus macaques

Evaluation parameters	Diversity index					
	OTUs	Chao1	Shannon	Simpson	Faith’s PD	Good’s coverage
Rhesus macaques	246±40	264.7±46.8	5.98±0.5	0.1±0.04	15.4±2.1	0.99±0.003
Cynomolgus macaques	266±53	297.7±64.2	5.38±0.6	0.05±0.03	18.2±2.8	0.98±0.004
Mann-Whitney U	0.41	0.18	0.001	<0.0001	0.0028	0.0047

### Beta diversity of rectal microbiota in rhesus and cynomolgus macaques

**Figure 1:**
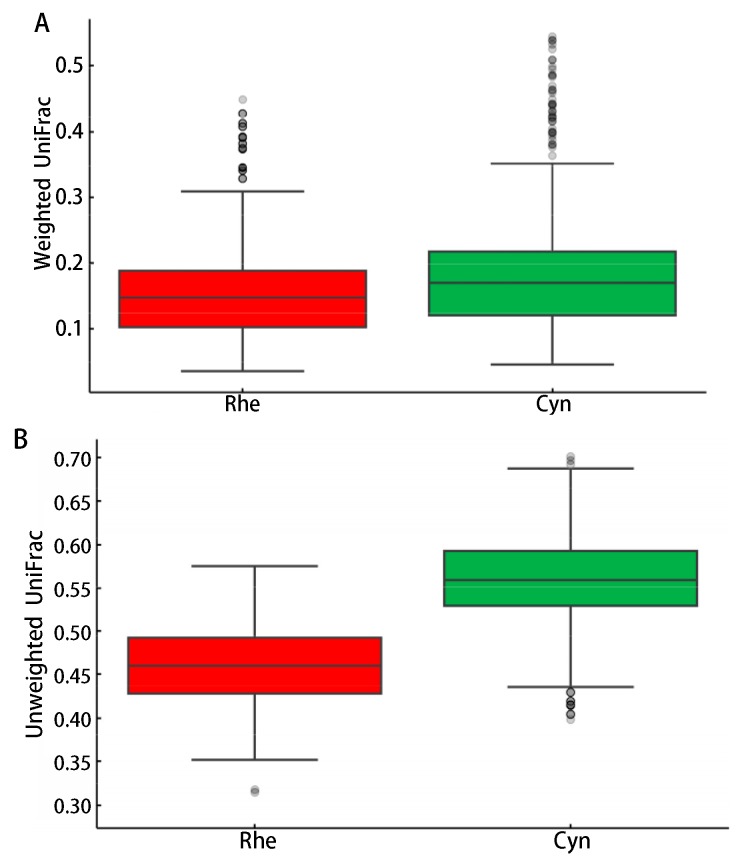
Differences in rectal microbiota beta-diversity between rhesus and cynomolgus macaques

To further reveal the differences between the two species, we examined the beta diversity (level of diversity or dissimilarity between samples) of the microbiota of the two species. As shown in Figure 1, the weighted and un-weighted UniFrac distances (Lozupone et al., 2011) of the microbiota were significantly different between the two species (*P*<0.05, Wilcoxon sum rank test).

Weighted (A) and unweighted (B) UniFrac distances are shown (Y-axis) for rhesus (Rhe) and cynomolgus (Cyn) macaques.

### Composition of phyla in the rectal microbiota from rhesus and cynomolgus macaques

**Figure 2:**
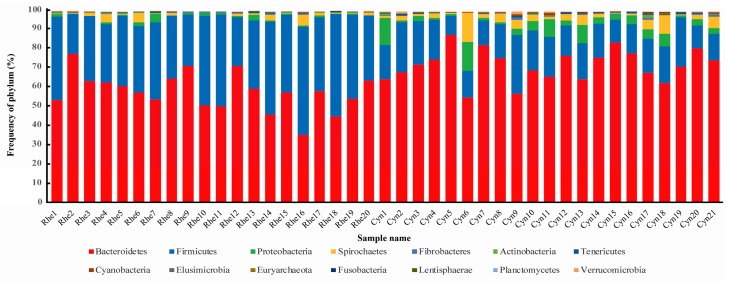
Phylum level taxon composition of the rectal microbiota of rhesus and cynomolgus macaques

As shown in Figure 2, Bacteroidetes, Firmicutes and Proteobacteria were the dominant phyla in both rhesus and cynomolgus macaques, representing more than 95% of rectal microbiota. Spirochetes was also present, although relatively rare. Bacteroidetes and Proteobacteria were significantly more abundant in the rectal microbiota of cynomolgus macaques than that of rhesus macaques, whereas Firmicutes was significantly more abundant in rhesus macaques than in cynomolgus macaques (*P*<0.05, Mann-Whitney *U* test).

Frequency of microbial phyla (Y-axis) is shown for rhesus (Rhe1-Rhe20) and cynomolgus (Cyn1-Cyn21) macaques. Color code for each phylum is shown at the bottom of the image.

### Composition of genera in the rectal microbiota from rhesus and cynomolgus macaques

**Figure 3:**
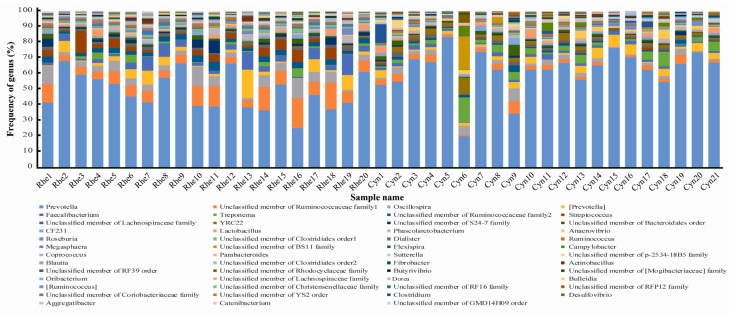
Genus level taxon composition of the rectal microbiota of rhesus and cynomolgus macaques

The dominant genera of the microbiota of the two species were *Prevotella*, *Ruminococcaceae*, *Oscillospira*, *Faecalibacterium* and *Treponema*, with *Prevotella* being the most abundant (Figure 3). *Prevotella* and *Treponema* were significantly more abundant in cynomolgus macaques than in rhesus macaques, whereas *Ruminococcaceae*, *Oscillospira* and *Faecalibacterium* were significantly more abundant in rhesus macaques than in cynomolgus macaques (*P*<0.05, Mann-Whitney *U* test).

Frequency of microbial genera (Y-axis) is shown for rhesus (Rhe1-Rhe20) and cynomolgus (Cyn1-Cyn21) macaques. Color code for each genus was shown at the bottom of the image.

### Metagenome functional content in the rectal microbiota of rhesus and cynomolgus macaques

**Figure 4:**
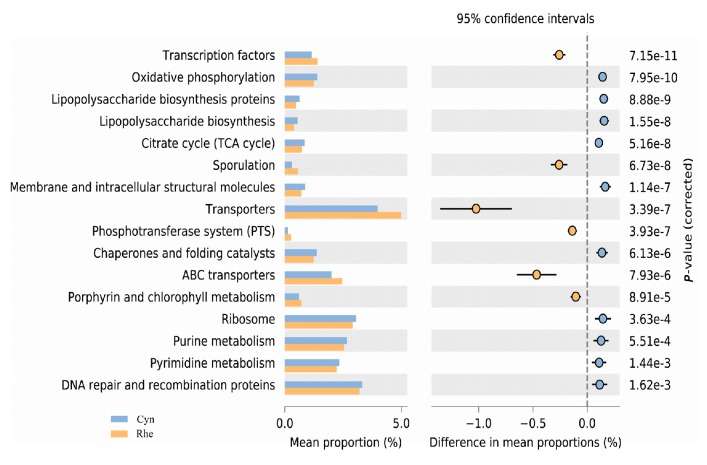
Functional contents predicted in the rectal microbiota of rhesus (Rhe) and cynomolgus (Cyn) macaques

As shown in Figure 4, various functional components in the rectal microbiota were significantly different between rhesus and cynomolgus macaques. More transcription factor genes were predicted in the rectal microbiota of rhesus macaques compared with cynomolgus macaques, whereas more genes related to oxidative phosphorylation, lipopolysaccharide (LPS) biosynthesis proteins, lipopolysaccharide biosynthesis and citrate cycle (TCA cycle) were predicted in cynomolgus macaques.

Proportion of functional components in the rectal microbiota is shown for rhesus (blue bar) and cynomolgus (orange bar) macaques. Image was generated with STAMP software using data from PICRUSt analyses.

## DISCUSSION

Rhesus and cynomolgus macaques are frequently used in studies of human diseases, in particular those associated with abnormality of gut microbiota such as diabetes and AIDS (Bauer et al., 2011; Dillon et al., 2016; Letvin et al., 1985). However, different results have been reported using the two species; for example, less severe levels of lymphopenia and lower set-point viral loads have been observed in simian immunodeficiency virus (SIV)-infected cynomolgus macaques in comparison to that in SIV-infected rhesus macaques, and cynomolgus macaques survive longer following SIV infection than rhesus macaques (Reimann et al., 2005; ten Haaft et al., 2001). In the current study, we compared the composition and functional components of the rectal microbiota of these two species by deep sequencing of the 16S rRNA gene and bioinformatics analyses. Results indicated that although Bacteroidetes, Firmicutes and Proteobacteria were dominant in both species, their relative abundances were significantly different.

Metagenomics prediction found many differences between the rectal microbiota of the two species, including differences in lipopolysaccharide (LPS) biosynthesis proteins and LPS biosynthesis. These differences in LPS were consistent with the differences in the abundance of bacterial taxa, i.e., more gram-negative phyla (Bacteroidetes and Proteobacteria) were found in cynomolgus macaques. Microbial translocation and attendant immune activation and systemic inflammation can drive rapid disease progression in HIV/AIDS patients. More LPS in the gut lumen generated by microbiota indicates higher basal levels of LPS, which can translocate if the mucosal barrier is damaged. Pig-tailed macaques (*Macaca nemestrina*) have been shown to progress to AIDS more rapidly than rhesus macaques after SIV infection, which may be associated with the higher basal levels of LPS translocation in pig-tailed macaques than rhesus macaques (Klatt et al., 2010). However, whether the differences in rectal microbiota between the two species are linked to the different results observed between SIV infected rhesus and cynomolgus macaques needs further study.

Microbial composition of gut microbiota can be shaped by both the host and environment (Rawls et al., 2006). Complex interacting factors such as diet, hygiene, environmental contact, antibiotic use and breastfeeding can influence microbiota composition in early life (Kaiko & Stappenbeck, 2014). The two closely related species used in this study were fed the same monkey chow and housed in the same experimental animal center. Although microbial species richness was not significantly different, as shown by the Chao 1 index and the number of observed OTUs, the alpha diversity of the rectal microbiota was significantly different between the two macaque species when the evenness and phylogenetic relationships of the microbial species were considered (Table 1). Furthermore, the beta diversity, taxon composition and functional content of the rectal microbiota were also significantly different between the two species (Figure 1, Figure 2, Figure 3 and Figure 4). Therefore, our data demonstrated significant differences in the microbial composition and function between the two species. It seems informative to dissect the factors that may have caused these microbiome differences, both host and environmental factors using these animals.

Although we analyzed taxa at the species level, only a small proportion of OTUs could be assigned to species. Among the top 10 dominant OTUs, *Prevotella copri*, *Faecalibacterium prausnitzii*, *Prevotella stercorea* and *Streptococcus luteciae* were the first, sixth, seventh and tenth most dominant species. Further studies are needed to identify the other OTUs to the species level. However, intestinal *Prevotella copri* is correlated with enhanced susceptibility to arthritis (Bernard, 2014) and *Faecalibacterium prausnitzii* is identified as an anti-inflammatory commensal bacterial in Crohn’s disease patients (Sokol et al., 2008). Thus, the presence of these commensals qualifies rhesus and cynomolgus macaques as suitable animal models for studies related to these bacteria.
